# Are We Ready to Build a System for Assisting Blind People in Tactile Exploration of Bas-Reliefs?

**DOI:** 10.3390/s16091361

**Published:** 2016-08-24

**Authors:** Francesco Buonamici, Monica Carfagni, Rocco Furferi, Lapo Governi, Yary Volpe

**Affiliations:** Department of Industrial Engineering, University of Florence, Florence 50139, Italy; rocco.furferi@unifi.it (F.B.); monica.carfagni@unifi.it (M.C.); lapo.governi@unifi.it (L.G.); yary.volpe@unifi.it (Y.V.)

**Keywords:** hand-tracking system, Kinect sensor, 3D reconstruction, blind people

## Abstract

Nowadays, the creation of methodologies and tools for facilitating the 3D reproduction of artworks and, contextually, to make their exploration possible and more meaningful for blind users is becoming increasingly relevant in society. Accordingly, the creation of integrated systems including both tactile media (e.g., bas-reliefs) and interfaces capable of providing the users with an experience cognitively comparable to the one originally envisioned by the artist, may be considered the next step for enhancing artworks exploration. In light of this, the present work provides a description of a first-attempt system designed to aid blind people (BP) in the tactile exploration of bas-reliefs. In detail, consistent hardware layout, comprising a hand-tracking system based on Kinect^®^ sensor and an audio device, together with a number of methodologies, algorithms and information related to physical design are proposed. Moreover, according to experimental test on the developed system related to the device position, some design alternatives are suggested so as to discuss pros and cons.

## 1. Introduction

Lack of sight affects blind people’s possibilities in many aspects of everyday life. Movements, tasks and actions that are simple, or even trivial, to sighted people become really challenging for blind people (BP). To support BP in a great number of situations, in the last decades many devices have been designed all over the world. In most cases, research has been focused on developing systems for assisting BP in their everyday activities such as walking [1], reading books, using computers [2] and so on. However, there are other blind people needs that, although not essential for living, contribute to the overall well-being of an individual. The possibility of enjoying artworks is probably one of the most relevant one since it helps BP in taking part, on an equal basis with others, in cultural life. Not surprisingly, some museums (e.g., the Omero Tactile Museum of Bologna or the Art Institute of Chicago) have created tactile exhibitions dedicated to blind people. The majority of museums, however, present touchable reproductions of sculptures or other 3D objects; tactile 3D reproductions of pictures or other 2D artworks are very rare and only a few institutions have them at their disposal. Usually, these models are handmade by artists thus offering artistic 3D interpretations of the 2D original artwork. To increase and speed-up this “translation” process, in the last few years a few computer aided approaches have been developed [3,4,5]. Recent studies [6,7] suggest that the mere tactile exploration of 3D models (even in case these are optimally reproducing the original painting) is not sufficient to fully understand, and enjoy, the artwork. Blind people understanding of the original artwork is, in fact, subject to a lot of factors (e.g., sensitivity and personal ability of the person, size and quality of the tactile model); for this reason a good quality verbal guidance is essential in order to appreciate even the best possible artistic relief reproduction of a given painting. Such a verbal description is usually provided by a sighted person (museum employee, accompanying person, etc.); this allows the blind person to build a complete mental image of the tactile model, and to not be stopped by the lack of comprehension of a single element of the bas-relief. The presence of another person, however, could be perceived as a limiting factor in enjoying the artwork since the blind person is forced to discover in the perspective of someone else; art is through a language that requires autonomy and freedom to be fully apprehended!

The introduction of an automatic verbal guide could increase the autonomy of the user during the exploration, allowing him to lead the experience (e.g., autonomously establishing the time needed for a full appreciation, moving the hands freely, taking time to think, etc.), achieving the same freedom of sighted people. To fulfil this goal, the guide should not be merely automatic (e.g., audio-guide such as the ones already available for sighted people), but rather “active” i.e., capable of following the user’s movements so as to provide information in form of verbal descriptions [7].

This ambitious objective is still far to be accomplished in scientific literature, not only for technical restrains: guiding a BP in the exploration of an artworks cannot be limited to a description of an artwork scene and/or of touched areas, but is rather a gradual help to acquire information and to organize it into a “mental scheme” that become progressively more and more complete and detailed. However, the design of a first-attempt system able to automatically provide verbal information of touched areas is still an advancement of the state of the art in this topic.

With this aim in mind, the authors of the present work presented a brief feasibility study [8] of a system to improve blind people tactile exploration of bas-reliefs, where a possible methodology for conceiving an active guide for BP was sketched.

Starting from such a preliminary work, the present paper provides a comprehensive description of the design phases required to build a first-attempt cost-effective system able to properly guide BP in exploring tactile paintings. Such a designed system consists of (1) a 3D Kinect^®^ sensor + software package to track the user hands; (2) a number of algorithms capable of detecting the position of the bas-relief in the same reference frame defined by the acquisition sensor; (3) a number of algorithms aiming at detecting the position and the distance of the user hand/finger with respect to the model; (4) the complete knowledge of the digital 3D bas-relief model and (5) an appropriate verbal description linked to relevant objects/subjects in the scene. The designed system, integrating latest methodologies and algorithms, represents a first consistent step in building an assistive system (not obviously aimed in completely replacing human assistance) to help BP in tactile exploration.

The remainder of the paper is as follows: in Section 2 a brief description of the state of the art for most relevant previous works (related to the designed system) is provided. In Section 3 the system hardware layout is described. In Section 4 methods and algorithms implemented and tested to build the Kinect^®^ sensor-based system are provided. In Section 5, physical layout alternatives of the system are analyzed. Finally, conclusions and future works are discussed in Section 6.

## 2. Background

The bas-relief exploration system (BES) relies on the implementation of well-known pre-existing methods to perform hand tracking, point cloud registration and 3D evaluation of the distance between two point clouds. Therefore, it’s hereby presented a brief review of these techniques, focused on the most promising approaches in literature for the considered application.

### 2.1. Hand Tracking

Hand tracking (HT) techniques aim at identifying, in real-time, the 3D position of a human hand. This goal is tackled with various approaches in the state of the art, using different data inputs and strategies. HT has been extensively applied in a number of fields: gestural interfaces, virtual environments and videogames are only few examples of the areas where it is gradually becoming a key-factor [9,10,11,12]. Application of HT techniques to help impaired people, including BP, in a number of everyday life problems makes no exception [13,14,15]. Since for the present application the HT system should not limit the user’s haptic sensitivity and his/her gestural freedom (allowing for a fulfilling tactile exploration), among all the different HT techniques available in literature, this state of the art focuses on the vision-based ones. This class, in fact, uses only optical sensors (i.e., cameras, 3D optical scanners and other unobtrusive devices) to obtain data. Vision-based techniques can be roughly classified in two great groups: appearance-based [16,17,18] techniques and model-based ones [19].

Model-based approaches are the most interesting for the present application since provide a full DOF hand pose estimation together with real-time 3D position of the hand. In detail, a digital model of the hand, comprising all joints and articulations, is used. Usually, the solution is retrieved performing a minimization of an objective function that describes (using data from a set of visual cues) the discrepancy between observed data (real position of the hand) and the solution obtained using the digital model. Accordingly, although computationally costly, model-based approaches are the best candidates for this application where the hand position must be determined continuously and entirely.

A first approach to model-based tracking is presented in [20]; a 27 DOF hand modelled by quadrics is used to generate the contours of the hand, which are then confronted with processed images of the real hand. De La Gorce et al. [19] instead propose an approach that takes advantage of shading and texture information as visual cues to compare the digital model and data observed from a single RGB camera. One of the most promising works using model-based approach is the one proposed in [17] where a Microsoft Kinect^®^ is used to obtain 3D data from the scene.

The user hand in 2D and 3D is isolated from the background by means of a skin colour detection followed by depth segmentation. The hand model (palm and five fingers) is described by geometric primitives and parametrized encoding 26-DOF (i.e., is represented by 27 parameters). The optimization procedure is carried out by means of a Particle Swarm Optimization technique [21]. The procedure contemplates temporal continuity of subsequent frames, searching for a solution in the neighborhood of the one found for the last frame analysed. The authors further developed their work in [22,23,24], covering simultaneous tracking of two hands and tracking of a hand interacting with real objects.

### 2.2. Point Cloud Registration

As widely recognized [25,26,27], point cloud registration is a class of algorithms that perform the alignment of two partially or entirely overlapping sets of points by means of a roto-traslation, minimizing relative distances. Among the wide range of methods for point cloud registration, the present work focuses on rigid techniques i.e., the ones that perform the alignment of the two sets of point by means of a rigid transformation (without changing the relative position of the points belonging to the transformed point cloud).

Rigid registration is usually performed by means of a two-step procedure: a first coarse registration and a subsequent fine one. Coarse registration performs a rough alignment of the two point sets, minimizing the distance between correspondences, such as points, curves or surfaces (or other geometric entities) extracted from the dataset with different criteria [27]. A number of algorithms can be used to perform coarse registration: Point Signature, Spin Image, RANdom SAmple Consensus (RANSAC)-based, Principal Component Analysis (PCA) and genetic algorithms. Fine registration, on the other hand, uses the result obtained by coarse registration as starting point and searches, in its neighborhood, for a more refined solution.

Among the wide range of algorithms available in the scientific literature, the most relevant for the present work are: the Iterative Closest Point (ICP) (which has been implemented in many different ways in recent years), the Chen’s method (a variation of ICP), the signed distance fields and genetic algorithms [28]. ICP and Chen’s methods are, by far, the most common and used: presented at the beginning of 90s, such methods are now implemented in many software libraries.

The ICP method aims to obtain an accurate solution by minimizing the distance between point-correspondences, known as closest point. When an initial estimation is known, all the points are transformed to a reference system applying the Euclidean motion. Then, every point in the first image is taken into consideration to search for its closest point in the second image, so that the distance between these correspondences is minimized, and the process is iterated until convergence. Chen’s method is quite similar to ICP; the only difference is the use of point-to-plane distance instead of point-to-point.

The minimization function is defined by the distances between points in the first image with respect to tangent planes in the second. In other words, considering a point in the first image, the intersection of the normal vector at this point with the second surface determines a second point in which the tangent plane is computed. The algorithm is, in this formulation, usually less conditioned by local minima and by the presence of non-overlapping regions [27].

### 2.3. Distance Evaluation

A number of methods coping with distance evaluation between sets of 3D points can be found in the literature [29,30,31,32]. Specifically, this work deals with the so called nearest neighbour search (NNS) problem, (also known as “proximity search”), which addresses the goal of finding the nearest point, within a data set, to a given query point (and the consequent computation of its distance). Although very simple in its definition, this issue becomes complicate either when the data set consists of a huge number of points or when a high number of query points are provided. Due to its importance in a number of computer vision problems, over the years the NNS problem has been tackled with several different strategies, partially discussed in this section.

Basically, NNS methods exploit the construction of search trees among the inspected dataset (i.e., data structures that organize the information about points distribution in a convenient way), in order to increase the efficiency of the nearest point search. In one of the most used methods, i.e., the “KD-Tree” one [29] a k-dimensional tree-like structure is created by means of recursive binary partitions of the dataset resulting from regions circumscribed by k-dimensional hyper-planes.

Another known method is the so called “Ball Tree” (also known as “Metric Tree”): in this case, the dataset is described by a tree modelled using hyper-spheres; this kind of structure, although computationally costly to build, guarantees a faster search, especially with high-dimension problems.

## 3. System Layout

**Figure 1:**
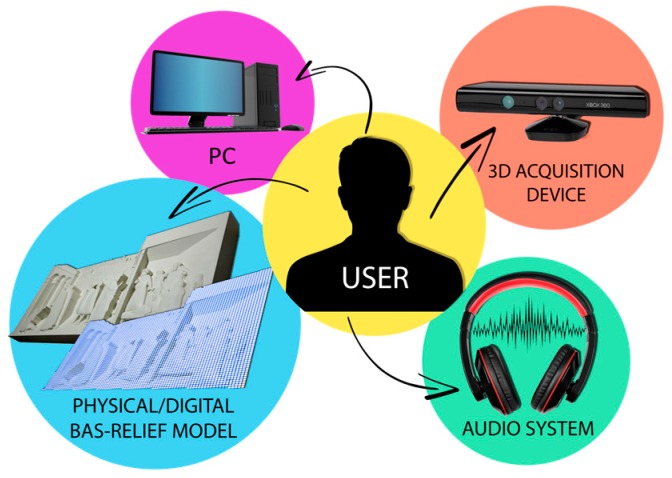
BES layout.

As depicted in Figure 1, the layout of the designed BES consists of:

(1) A physical bas-relief to be explored by BP and its digital counterpart (e.g., a high-definition point cloud/polygonal model describing it).
  Even if, in principle, any kind of bas-relief could be used for developing the BES, in this work the used tactile models are the ones created by using the procedure described in [33], where shape from shading-based methods are devised to obtain both 3D polygonal models (e.g., STL) and a physical prototype of such a digital model starting from a shaded picture (for example a renaissance painting). In fact, by using such a procedure both the physical and digital 3D information are directly available. In any case, the proposed procedure can be applied to any kind of bas-relief (or in case the bas-relief is not allowed to be touched, to a replica) since the required initial information (polygonal model) can be easily achieved using a commercial 3D scanner.
(2) A 3D acquisition device capable of (i) tracking the user hands and (ii) detecting the position of the physical bas-relief in its reference frame.
  The device used to build the system is the Microsoft Kinect^®^. As widely known, it consists of a projector-camera triangulation device furnished with a 43° vertical by 57° horizontal field of view that covers, at 1 m distance a visible rectangle of 0.8 m × 1.1 m. Such a field of view, to be considered as a plausible value for tracking according to [17], is required to cover the typical dimension of tactile bas-reliefs.
(3) A PC workstation, in control of the whole BES.
  This element is responsible for the hand tracking, the required calculations (point clouds registration and distances computation, as previously described) and for the touch identification. The hardware needs to be equipped for GPU computing, and with hand tracking performances comparable with [17], to assure satisfying results.
(4) An Audio system.
  Since the final outcome of the BES is, as already mentioned above, a verbal description of the scene and/or of touched objects or features, the system is equipped with headsets/headphones. Of course, to locate headsets could be difficult for unaccompanied BP; unfortunately, since the installation is specifically addressed to museum installations, the use of audio speakers could not represent a valid option.

## 4. Materials and Methods

This section provides a step-by-step description of methods and algorithms implemented and tested to build such a system. All necessary procedures were developed using Matlab^®^ that offers a number of embedded tools and algorithms useful for this application.

**Figure 2:**
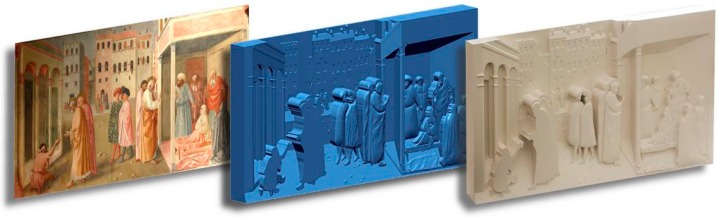
“Guarigione dello storpio e resurrezione di Tabita” by Masolino da Panicale: original artwork, digital 3D model and 3D-printed bas-relief.

To help in understanding the devised system the overall method is described with reference to the tactile reproduction of ”Guarigione dello storpio e resurrezione di Tabita” by Masolino da Panicale (see Figure 2). The physical model has size 900 × 420 × 80 mm while its digital counterpart is described by 3.6 million points.

### 4.1. Hand Tracking

The first step of the entire procedure consists of detecting the position of the bas-relief to be explored in the Kinect^®^ reference frame and, contextually, to detect the areas touched by the user (whose hand position has to be expressed in the same reference system). In fact the knowledge of the position of the hand, together with the position of the tactile model, will allow to determine if the user is touching the bas-relief and in which area. This information, however, must be known in the same reference system.

Accordingly, the very first step of the proposed procedure consists of tracking the user hand. Among the several interesting works in literature exploring the use of the Kinect^®^ sensor as a device for hand tracking [10] the HT system used for building the proposed system is the one developed in [17]. Such a real time HT system, working using Microsoft Kinect^®^ as optical sensor, is characterized by a 20 fps framerate when running on modern architecture PCs and moreover it does not require any visual marker on the user’s hand. Moreover, the system is delivered with a convenient ready-to-use library (developed by the Forth Institute in 2015). Accordingly, the use of the above mentioned HT system is a straightforward method to know the fingertip position directly in the acquisition device reference system. For the proposed application a high frame rate value is crucial. In fact, it directly affects the quality of the solution provided by the system: with higher frame rate values rapid movements of the hand are more easily registered. Moreover, a small time step between evaluated solutions increases the soundness of the last-known position, used as reference value for the location of the hand.

For this reason, in the proposed system, the procedures accomplishing the HT (i.e., the HT library), have been left free to run separately and independently to the rest of the algorithms (e.g., touch identification), which could slow down the HT. In fact, two main cycles run simultaneously: a hand tracking cycle (HTC), which evaluates continuously hand pose solutions and saves them, and a touch identification cycle (TIC). TIC consists of a number of procedures (extensively described in next steps) that rely on the latest hand pose solution stored in the system by the HTC to assess if and where the user is touching the bas-relief.

Moreover, to reduce the complexity of tracking problem, hand tracking has been performed with reference to a single fingertip (index). This choice is recommended for this application since the proposed BES is only a prototypal version of a future automatic verbal guide system to be installed into museum environments. Consequently, the final result obtained by using the HT system is to detect the coordinates of the extreme point of the index fingertip in the Kinect^®^ reference frame.

### 4.2. Bas-Relief Positioning

To retrieve the position of the tactile bas-relief in the Kinect^®^ reference frame, the simplest way is to use such a device as a sort of “traditional” 3D scanner; with a single placement a 3D scan of the scene in the Kinect^®^ field of view is accomplishable simply using Kinect^®^ Fusion library.

However, the quality of the Kinect^®^ 3D scan is not good enough to obtain detailed information about the bas-relief or to identify the contact with the finger; especially with a single placement, obtained 3D polygonal model have a low resolution and is affected by high noise. Moreover, the Kinect^®^ acquires the entire scene (not only the bas-relief), resulting in lot of undesired scan points or polygons. Nonetheless, despite the device provides low-definition (LD) scans almost useless for accurate reconstruction of the scene, the scanned points can be used as a provisional reference for registering the (available) high-definition (HD) model as explained in the next procedural step.

### 4.3. Registration

Once the LD model (correctly referred to the device reference frame) is available, the original high-definition model (HD) of the bas-relief is registered upon the LD one. With this strategy, a very refined 3D model correctly referenced in the Kinect^®^ frame can be obtained. The registration is accomplished by using a two-steps procedure: first a coarse registration is performed to roughly align and over-impose the HD onto the LD points (belonging to the point cloud or polygon vertices in case polygonal models are used). Then, a fine registration, using the rough results obtained in the coarse registration as initial guess, is made to increase the quality of points’ alignment.

#### 4.3.1. Coarse Registration

As described in the introductory session, several methods for coarse registration are available in literature. However, in the present work, it is performed with an appositely devised interactive procedure, taking advantage of the hand tracking system implemented to obtain the required initial rough alignment. In effect, traditionally coarse registration algorithms search for geometric correspondences in the two point sets. This procedure, instead, imitates the common “point and click” procedure for coarse registration that is usually implemented in reverse engineering software (where the selection of correspondences is done by the user itself).

**Figure 3:**
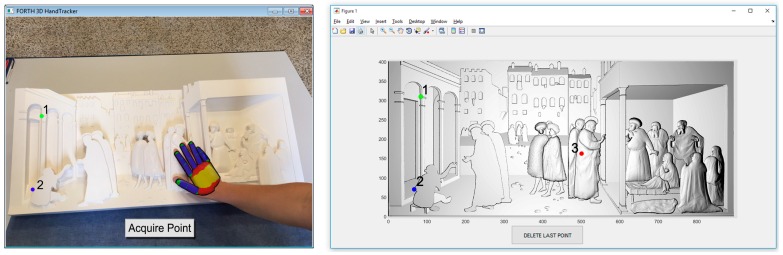
Custom-made coarse registration GUI.

In detail an appositely developed point-and-click interface (Figure 3) is used to pick a number N≥3 of non-aligned pairs of equivalent points in both the LD and HD clouds (or polygonal models).

To select the points in the LD model, the HT system is used as follows: first the user touches the desired point using the tracked fingertip. Once the contact between finger and physical bas-relief is established, the user click on the “acquire” pushbutton so that the coordinates of the fingertip are stored in a matrix $P_{\text{LD}}$ (size N×3) and a numbered tag is attached in the touched point. Subsequently, the user is required to touch the correspondent point on the HD model, using the sequence defined by the numbered tags. The coordinates of these points are finally stored in a matrix $P_{\text{HD}}$ (size N×3).

The $P_{\text{LD}}$ and $P_{\text{HD}}$ matrices, whose elements are the coordinates of, roughly the same points in, respectively, the reference frame of the LD and HD models, can be used for effectively registering the HD model onto the LD ones.

In fact, the reciprocal alignment between the two mentioned reference frames consists of the roto-translation described by the following equation:

(1) $$P_{\text{HD}}=R\times P_{\text{LD}}+t$$

where R is the rotation matrix and t is the translational vector. Since in Equation (1) both R and t are unknown, a proper procedure for determining them is required. In particular, a singular value decomposition (SVD)-based method (Besl and McKay, [34]), covering three steps has been used.

Firstly, the centroids of both sets of points $P_{\text{LD}}$ and $P_{\text{HD}}$ are computed as follows:

(2) $${\text{centroid}}_{\text{LD}}=\frac{1}{N}\overset{N}{\underset{i=1}{\sum }}P_{\text{LD}}^{i}$$

(3) $${\text{centroid}}_{\text{HD}}=\frac{1}{N}\overset{N}{\underset{i=1}{\sum }}P_{\text{HD}}^{i}$$

where $P_{\text{LD}}^{i}$ and $P_{\text{HD}}^{i}$ are the 1 × 3 vectors describing the coordinates of the ith point belonging, respectively, to the set $P_{\text{LD}}$ and $P_{\text{HD}}$.

Once the centroids are evaluated, it is possible to build the matrix H as follows:

(4) $$H=\overset{N}{\underset{i=1}{\sum }}\left(P_{\text{LD}}^{i}-{\text{centroid}}_{\text{LD}}\right){\left(P_{\text{HD}}^{i}-{\text{centroid}}_{\text{HD}}\right)}^{T}$$

The widely known SVD procedure can now be applied to matrix H allowing to determine the matrices U, W and V:

(5) [U,W,V]=SVD(H)

As a consequence, the rotation matrix R is easily evaluable as follows:

(6) $$R={\text{VU}}^{T}$$

Once R is known, the translational vector can be evaluated using the following equation:

(7) $$t=-R\times {\text{centroid}}_{A}+{\text{centroid}}_{B}$$

Finally, the knowledge of R and t allows to determine the rough alignment between HD model and Kinect^®^ acquired LD one, according to Equation (2); in other words, to find the whole set of coordinates ${P^{\prime }}_{\text{HD}}$ of the HD model in the reference frame of the LD one (i.e., the Kinect^®^ reference frame) it is sufficient to apply Equation (1) as follows:

(8) $${P^{\prime }}_{\text{HD}}=R\times P_{\text{HD}}+t$$

This procedure showed good results on the point registration, especially when the points chosen by the user are well-separated and non-aligned.

#### 4.3.2. Fine Registration

Fine registration is performed starting from the roughly aligned point sets resulted from Section 4.3.1 (Coarse Registration). Among the already mentioned algorithms proposed in scientific literature, ICP and Chen’s method were tested so as to find the best one suited for this application.

Both methods perform an iterative minimization of properly defined distance functions. Given the two point sets ${P^{\prime }}_{\text{HD}}$ and $P_{\text{LD}}$ to be aligned, ICP iteratively searches for each ${P^{\prime }}_{\text{HD}}^{i}$ point of set ${P^{\prime }}_{\text{HD}}$ the nearest point $P_{\text{LD}}^{i}$ of set $P_{\text{LD}}$ and apply to the original set ${P^{\prime }}_{\text{HD}}$ a proper roto-translation to minimize the distance between the two points. At the end of iterations (reached when a proper cost function is minimized) the set ${P^{\prime \prime }}_{\text{HD}}$ represents the best HD aligned model.

As mentioned in Section 2, Chen’s method is quite similar to the ICP one, with the difference of using point-to-plane instead of point-to-point distances. Also using this algorithm, the final result is the set ${P^{\prime \prime }}_{\text{HD}}$ describing the aligned HD model.

**Figure 4:**
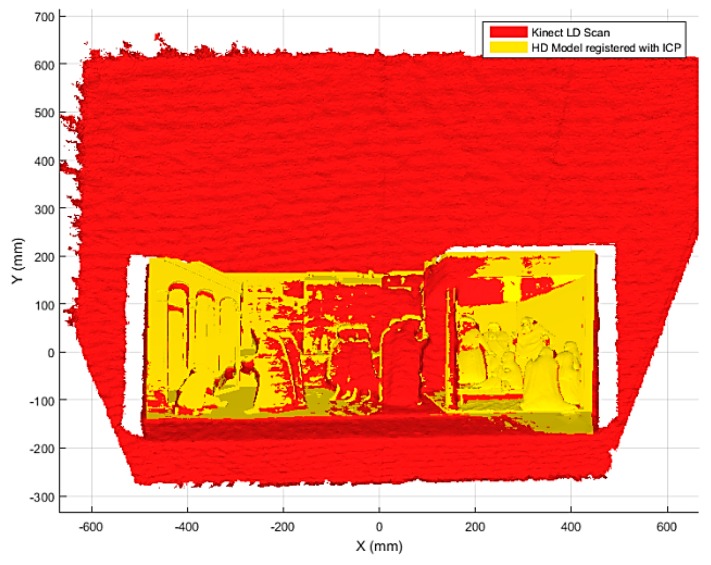
HD and LD bas-relief models after coarse and fine registration (ICP algorithm).

Both these methods, easily implementable in the Matlab^®^ environment, are reliable and show overall good results. Accordingly, ICP and Chen’s methods were tested on the registration of the LD and HD scans and showed comparable results. Despite Chen’s method being considered in the literature as the most reliable among the two analysed, for the proposed application tests demonstrated that it exhibit more sensitivity to local minima during iterations. Given that fine registration needs to be executed just once during the calibration of the models (i.e., before the bas-relief exploration starts), solution stability was considered as the most important factor. ICP was therefore chosen as preferred method to perform fine registration. Tests performed by authors demonstrated that the average time for convergence of ICP is in the range of 5−8 min, with model dimensions in the order of 10^5^ points for the HD scan and 10^6^ points for the LD scan (it has to be noticed that LD scans contains also points that are not belonging to the bas-relief). Iterations usually stop with a RMS error between 2−3 mm, value comparable with the Kinect^®^ accuracy. A visual example of the final result obtained with this method is depicted in Figure 4.

It is important to remark that the whole registration procedure (coarse + fine) should be performed only one time, before the exploration task starts, or at worst it has to be repeated in case the relative position between the bas-relief and the sensor changes for any reason.

### 4.4. Touch Identification

As already said, thanks to the strategies presented in Section 4.1, Section 4.2 and Section 4.3, the position of the index fingertip and of all the points composing the HD bas-relief model are known in the same reference system. The next step consists of identifying if and where the contact between the finger and the bas-relief occurs.

To perform touch identification, the most convenient method is to find, among all the points of the 3D model, the nearest to the fingertip. Moreover, the distance between such two points is compared to a given threshold to decide whether the finger is in contact or not with the bas-relief. To find the nearest point, the k-nearest neighbour algorithm method was tested against both the “N-D nearest point search” method and the “brute force” method.

Though the proposed prototypal application is based on a single query point (i.e., the index fingertip) algorithms were tested with up to 16 query points with the aim of simulating more complex versions of the system (i.e., with more hand points processed by the system at the same time and/or with more points taken in a single finger). In particular, tests were carried out increasing the number of query points (1–2–4–8–16 points) and the dimension of the dataset (50–100–200–400–800–1600 k points). K-nearest neighbour resulted as the best performing method in all the situations since its computing time is lower than 0.1 s even in the most challenging condition. Such a value guarantees a frame rate of approximately 10 fps and may be therefore considered acceptable for the touch identification task.

**Figure 5:**
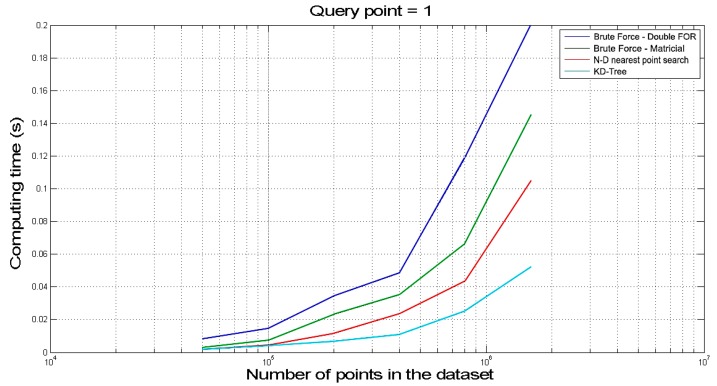
Nearest point methods comparison, 1 query point.

In Figure 5 the results of the test performed with one query point are presented. Computing time value, equal to about 0.05 s in the worst conditions, confirms that the implemented k-nearest neighbour algorithm performs perfectly for the considered application.

Accordingly, once the nearest point $P\in {P^{\prime \prime }}_{\text{HD}}$ to the query point Q is evaluated using the k-nearest neighbours (together with the distance value d) it is possible to identify the touching condition. In fact, if d is smaller than the threshold value $d_{\text{touch}}$, the finger is identified as in touch with the bas-relief; conversely, the devised algorithm considers that no contact occurred between the finger and the HD model. In this last case, the current touch identification cycle (TIC) is considered completed and the touch identification task starts again.

**Figure 6:**
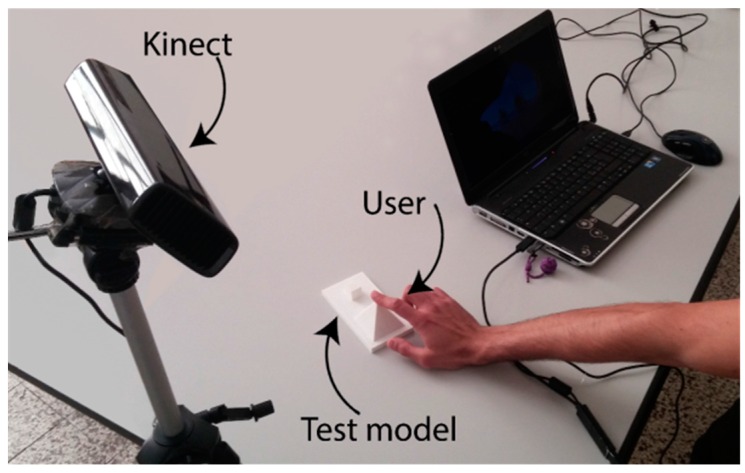
Hardware for the test of touch identification.

On the basis of a number of tests performed using the whole system of Figure 6, the threshold value $d_{\text{touch}}$ was set to 5 mm. This value showed the best compromise between false positive and negative occurrences.

A touch identification test was performed using the setup pictured in Figure 6 to evaluate the performances of this phase. The Kinect^®^ sensor was placed at a distance of 1 m from the test model (a plausible value to obtain good hand tracking results, according to [17]). The test began with the hand tracking calibration (required by the implemented method), which registered the hand model upon the user hand. Once that the tracking was stable, the user approached the test model with his hand, describing a roughly vertical movement, until his right index fingertip touched the tip of a pyramidal shape (i.e., the target point of the test), as in Figure 6. The test results (Table 1) showed 74 positive touch identifications on a total of 100 runs. Eighteen false negatives (situations where the touch condition was not recognized) occurred, partly caused by the complete loss of the tracking; eight false positives were registered, characterized by the identification of touch between the finger and the model that significantly anticipated the actual contact between the two.

These values, although promising for a first test, represent a significant limit to an actual implementation of the whole system; this aspect, therefore, needs to be considered and addressed during the study of a first BES functional prototype.

### 4.5. 3D Segmentation and Region Identification

The last step to be performed consists on the identification of the touched bas-relief region (in such a region, in the future, it will be possible to convey associated verbal description). Region identification starts from a 3D segmentation of the HD digital bas-relief model. Different regions of the bas-relief are identified considering their significance in the original artwork and according to the desired level of detail. These m regions are easily segmentable using a reverse engineering software (e.g., Polyworks^®^); for the proposed procedure each segment is individually stored as an STL file.

Of course each segmented region consists of a number of points of the set ${P^{\prime \prime }}_{\text{HD}}$. As a consequence it is possible to associate to each point of the 3D model a label (from 1 to m) identifying the region containing the point itself. In other words, it is possible to build a matrix A (size m×4) where the first three columns are the coordinates xyz and the last column is the associated label.

Once the touch identification cycle identifies the touching condition, the corresponding touched region is detected simply by searching in matrix A the label associated to the coordinates P.

### 4.6. Verbal Description

Each segmented region of the HD model can also be enriched by an audio file containing a verbal description; by a way of example it is sufficient to associate to each region a single wav file. Once the touched area is identified, such description can be transmitted to the user by means of a pair of headphones, to guide him/her in the exploration so to allow a full-immersion experience. Until the description is provided, the TIC is maintained in stand-by in order to avoid undesired interruptions due to a different position of the finger.

## 5. Physical Layout Alternatives

**Figure 7:**
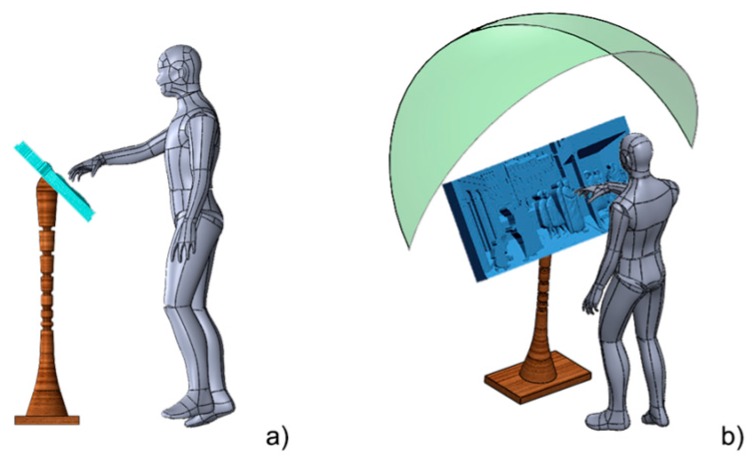
(**a**) CAD model of the bas-relief and the user; (**b**) CAD model of the plausible region (green) to host the Kinect^®^.

To select the best physical layout of the devised prototypal system, the relative positions among the user, the bas-relief and the Kinect^®^ have to be investigated. In fact, the system should be capable of providing the best possible accessibility to the tactile bas-relief thus maximizing the comfort during the tactile exploration and, at the same time should guarantee the best system performances. To this purpose, CAD models of the 50th percentile male and of the bas-relief to be explored have been realized in order to get a first idea of the overall dimensions of the two elements (see Figure 7a).

The bas-relief has been positioned at an approximate height of 1.2 m from the ground and with an inclination toward the user of 45°. This position was determined to be the most comfortable for the user, thanks to information gathered by the authors in tests performed together with a panel of blind persons, within the T-VedO project [33]. Starting from this configuration, it has been possible to determine the occlusions introduced in the scene by the users’ hand (a key information to place the visual sensor) and, therefore, to identify the areas suitable for housing other elements of the system.

Of course, the most important element to be positioned is the Kinect^®^; according to [17], and supported by further tests performed by authors of the present work using the device library, an average distance of 1 m between the sensor and the user’s hand may be considered among the best options to obtain good performances in terms of resolution and visibility. Moreover, besides the possible obstruction of the scene provided by users’ hand, it has to be taken into account that some areas have to be left free for accessing the bas-relief. As a consequence a portion of spherical shell with radius equal 1 m, centred in the barycentre of the bas-relief and with an angle of 60° (Figure 7b) has been found as a plausible area to host the Kinect^®^.

**Figure 8:**
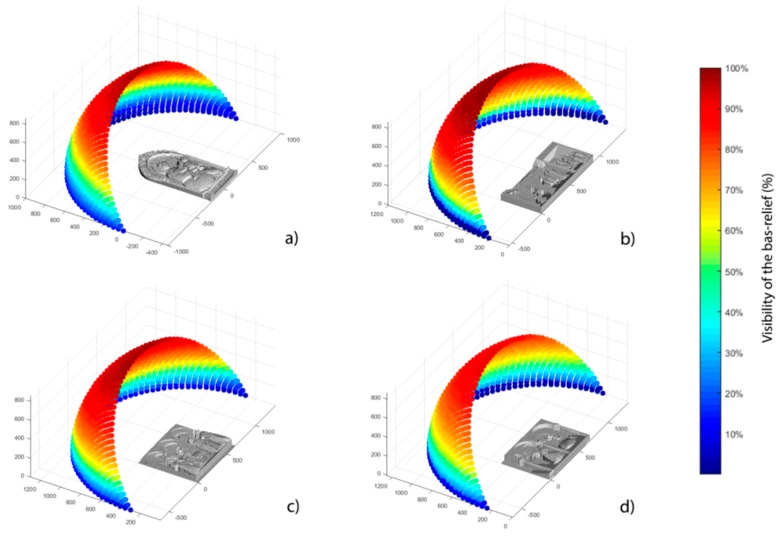
Visibility analysis for bas-reliefs; (**a**) “Madonna con Bambino e Angeli” by Niccolò Gerini di Pietro; (**b**) “Guarigione dello storpio e resurrezione di Tabita” by Masolino da Panicale; (**c**) ”Pala di Santa Lucia de’ Magnoli” by Domenico Veneziano; (**d**) ”Annunciazione” by Beato Angelico.

The sensor position influences also the percentage of bas-relief directly visible by the acquisition device due to the optical occlusions created by parts of the bas-relief itself. Since the system needs to be functional independently from the specific bas-relief selected for exploration, it is not possible to determine a sensor position for avoiding all possible self-occlusions. However, by studying a set of representative case studies it is possible to select a position which is generally suitable though not optimal for each single case. In detail, the percentage of bas-relief visible from a discrete set of points taken on the previously defined spherical shell is evaluated. The results, depicted in Figure 8, show a common central region that maximises the visibility percentage (with an average value near to 90%).

**Figure 9:**
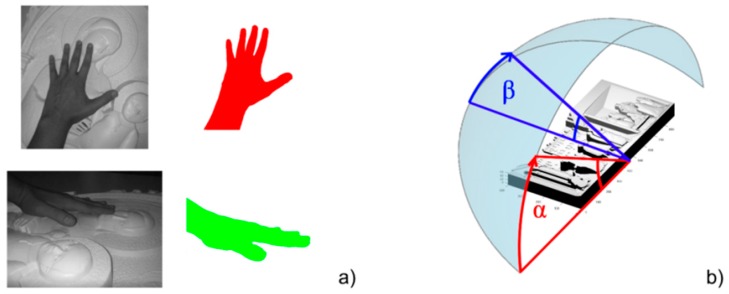
(**a**) Views and silhouettes of the user hand as seen by the Kinect^®^ with different elevation angles; (**b**) Bas-relief reference frame for Kinect^®^ positioning, where α is the azimuth angle and β the elevation angle.

To choose, among the set of positions that maximise the visibility of the bas-relief, the better solution, it has to be considered that the HT precision strongly depends also by the portion of users’ hand acquired during the sensor acquisition. Test performed with different positions of the Kinect^®^ (in the points with maximum percentage of visibility) showed that the best performance is obtained when the hand is placed perpendicular with respect to the line of sight of the Kinect^®^ sensor (see the red hand in Figure 9a). This is probably due to the fact that, in this position, the hand shows to the sensor its most distinctive features (e.g., the silhouette shows all five fingers and the back of the hand is clearly visible). Conversely, when the acquisition device line of sight is inclined with respect to the hand (see the green hand in Figure 9a), it shows less features and accordingly the silhouette could be lost or ignored during skin segmentation.

Since on the basis of the mentioned tests performed by authors in the T-VedO project, BP usually explore bas-reliefs with palms placed approximately parallel to the explored surface, among the possible configurations showing better visibility results, the preferable options are the ones where the sensor is placed approximately perpendicular to the bas-relief. Finally, it is possible to state that the best positioning option, referring to Figure 9b, corresponds to the angles α=90° (azimuth angle) and β=60° (elevation angle). In fact this configuration is the optimal compromise between bas-relief visibility and hand features recognition.

**Figure 10:**
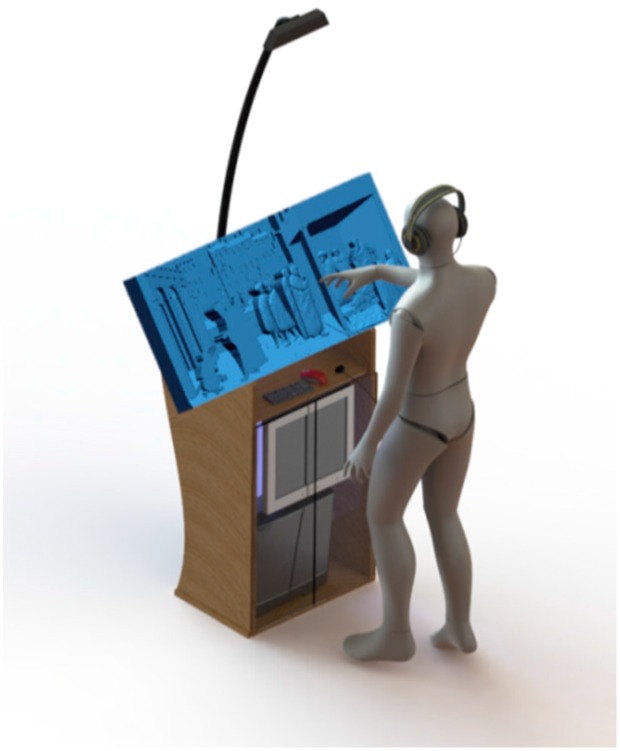
Final layout of the BES prototype.

On the basis of the above considerations, the final layout of the proposed prototype is the one depicted in Figure 10 where a vertical structure, located directly under the bas-relief, is designed with the aim of (1) containing the computer hardware and control devices; (2) sustaining the tactile model in the desired position; (3) placing the acquisition sensor in the above mentioned optimal position. The structure, moreover, has a lectern-like shape, suitable for museum exhibits.

## 6. Discussion and Conclusions

In this work, a full description of a first-attempt tactile bas-relief exploration system to improve blind people tactile exploration of artwork has been presented. The system, today in the form of prototype, consists of a bas-relief, a 3D scanner (Microsoft Kinect^®^) tracking the user’s hands connected to a PC, and an audio device providing the user verbal descriptions in response to the hand movements relatively to the bas-relief.

The system functionalities are of course limited, mainly due to the fact it tracks only a single finger and the verbal guidance is only an embryonal idea. Moreover, the accuracy of the touch identification needs to be carefully improved and assessed. The main drawback of the proposed system, up-to-date, is related to the HT system; in fact while slow movements and limited hand rotations are excellently tracked, the hand position is lost from time to time when movement speed increases. Accordingly, at this time it is still premature to think of an implementation of the entire system at least until the issues related to HT and touch identification are coped with. In other words, the answer to the question posed at the beginning is: “probably not yet!”.

Fortunately, additional refinements of the work in [22,23,24] are now under further development at the Forth Institute and the release of better HT algorithms is expected soon. Consequently, such improvements will be tested with the proposed system. Furthermore, the use of the new version of the Kinect^®^ (Kinect^®^ 2.0) with enhanced depth fidelity could improve the HT performance as well, together with an expectable higher resolution provided by the sensor.

Future work will be addressed to the implementation of the HT system to detect more fingers and even the two full hands. The introduction of multiple points, although challenging, could be useful to refine the identification logic of the regions (e.g., interpretation of the full hand position could resolve conflicts in the identification of two contiguous regions). Other issues that will be investigated to improve the system performances are: (a) lighting condition on the scene, which could affect skin segmentation performed by the HT system; (b) implementation of multiple visual sensors, to increase the number of viewpoints and strengthen the HT; (c) study of multi-cue strategies to increase the robustness of the hand identification by HT, appositely devised for this application and its features (e.g., computer vision techniques like background subtraction). Moreover, despite the fact the devised system is largely based on prior studies with a panel of BP, further studies will be assessed by involving more BP to highlight strengths and weaknesses of the proposed system as well to find possible improvements. Moreover, a detailed analysis of system performance on a panel of BP is still required to assess the effectiveness of the designed solution.

The implementation of a gestural interface (i.e., conferring different meanings to specifics gestures made by the user) could dramatically increase the autonomy of blind people, the interactivity of the system and, therefore, its potentiality. Different positions of the hand during exploration could, in effect, be interpreted to transmit different kind of information to the user (e.g., art-related, semantics of the touched regions). This particular issue is not trivial and accordingly more work is required prior to reach exploitable results. Issues such as cost of the entire system, industrial production feasibility, optimization of procedures at industrial scale constitute further future studies to be confronted with prior to effectively introduce the proposed system in museum environments.

## Figures and Tables

**Table 1:** Touch identification test results.

Number of Tests	Positive Touch Identifications	False Negatives	False Positives
100	74	18	8
